# Undernutrition and associated factors among adolescent girls in Diga District, East Wollega Zone, Ethiopia

**DOI:** 10.1371/journal.pone.0310225

**Published:** 2024-10-29

**Authors:** Emebet Bobo, Haile Bikila, Wandimu Muche Mekonen, Meseret Belete Fite, Gurmessa Enkossa Ayana, Werku Etafa

**Affiliations:** 1 Department of Pediatrics & Neonatal Nursing, Institute of Health Sciences, Wollega University, Nekemte, Ethiopia; 2 Department of Public Health, Institute of Health Sciences, Wollega University, Nekemte, Ethiopia; 3 Department of Internal Medicine, Institute of Health Sciences, Wollega University, Nekemte, Ethiopia; Zagazig University Faculty of Human Medicine, EGYPT

## Abstract

**Background:**

Undernutrition is a significant challenge for adolescent girls globally due to biological, social, and cultural factors. The consequences of undernutrition for adolescent girls are extensive, impacting their cognitive abilities, reproductive health, susceptibility to chronic diseases in later life, and the economies of nations. However, there needs to be a more comprehensive understanding of the nutritional status of adolescent girls in the Diga district, Ethiopia.

**Objective:**

This study aimed to assess the prevalence of undernutrition and its associated factors among adolescent girls in the Diga District, East Wollega Zone, Ethiopia.

**Methods:**

The study employed a community-based cross-sectional study design in Diga District, Ethiopia. Data was gathered from 651 study participants using a systematic random sampling technique, from July 10^th^ to August 10^th^, 2023. Data analysis involved using Epi-Data 4.6 and SPSS version 26 for data entry and statistical analysis, respectively. The Body Mass Index (BMI) and Height-for-age (HFA) Z-score are generated using WHO AnthroPlus version 1.0.4 software. Descriptive statistics and binary logistic regressions were used for data summarization and analysis, with significance testing at a p-value <0.05.

**Results:**

In this study, 16.3% (95% CI: 13.5–19.3) of adolescent girls were stunted, while 18.5% (95% CI: 15.4–21.5) were thin. Lack of access to health and nutrition information (AOR = 3.36, 95% CI: 1.38–8.23), limited crop variety within household’s crops (AOR = 1.66, 95% CI: 1.03–2.65), and household food insecurity (AOR = 2.76, 95% CI: 1.49–5.11) were factors associated with stunting. Poor dietary diversity scores (AOR = 7.52, 95% 95% CI: 2.92–19.39) and household food insecurity (AOR = 3.69, 95% CI: 1.96–6.93) were significantly associated with thinness.

**Conclusion:**

Conclusively, there was a low prevalence of stunting and thinness among adolescent girls in the Diga district, Ethiopia. Interventions aimed at enhancing household-level crop diversity, improving food security, providing adequate health and nutrition information, and promoting income-generating activities for adolescent girls can play a crucial role in improving their access to nutritious foods and healthcare, ultimately leading to better nutritional outcomes.

## Introduction

According to the World Health Organization (WHO), individuals aged between 10 and 19 years old are classified as adolescents [1]. Based on their age, adolescents are categorized into three stages: early (10–13 years), middle (14–16 years), and late (17–19 years). Globally, there are about 1.3 billion adolescents, constituting 16% of the world’s population, with approximately 90% living in low- and middle-income countries (LMICs) [2]. In Ethiopia, adolescents and children make up 48% of the total population, with adolescent girls representing 25% of this group [3].

Adolescents, particularly, girls, are often underestimated and challenging to assess, making it difficult to address their specific needs [4]. Throughout adolescence, individuals attain 15 to 25% of their adult height, up to 50% of their adult body weight, and achieve 40–60% of their peak bone mass [5]. Malnutrition among adolescent girls is a significant global public health issue [5, 6]. Undernutrition pertains to a condition where an individual’s intake of energy and essential nutrients is inadequate to meet their body’s requirements for optimal health and functioning [7].

According to a UNICEF report, the global prevalence of underweight among adolescent girls is 8% [2]. A study conducted in South Asia revealed that 40% of adolescent girls are undernourished [8]. In East Africa, a study showed that 16.50% of late adolescent girls experience undernutrition [9]. The 2016 Ethiopia Demographic and Health Survey (EDHS) report indicated that 29% of adolescent girls were classified as thin [3]. Moreover, being underweight or thin among adolescents is more common in rural areas of low- and middle-income countries (67%) than in urban areas [10]. Well-nourished adolescents are more capable of effectively utilizing their skills, abilities, and energy in the present, while also developing into healthy and responsible citizens and future parents of healthy offspring [11].

Undernutrition can have short-term and long-term consequences due to inadequate nutrition or as a secondary effect of illnesses [12]. Approximately 1.1 million adolescents die globally each year, with about 45% of these deaths attributed to malnutrition, which disproportionately impacts LMICs [13]. Adolescent girls are particularly at risk, especially in underdeveloped nations where early marriage is prevalent, leading to higher rates of reproductive morbidity and mortality [14]. Stunted and underweight adolescent girls are associated with diminished early growth, which is a primary factor in childhood mortality, contributing to around 12% of childhood deaths worldwide [15].

Undernourished adolescent girls are more likely to give birth to babies with health and nutrition issues, such as cognitive impairments, low birth weight, small size for gestational age, premature birth, poor bone development, weakened infection resistance, a higher lifetime risk of disease and mortality, and intergenerational cycles [12, 15–17]. Undernutrition in teenagers perpetuates an intergenerational cycle of malnutrition. Adolescent undernutrition is mainly caused by food insecurity, family size, and educational status of the family, meal frequency, dietary diversity level, adolescent health status, and hygiene related factors [18–29].

Despite the inclusion of adolescent nutrition services in the Sustainable Development Goals (SDGs), the nutritional status of adolescent girls has not significantly improved [2]. The Government of Ethiopia has implemented programs like the National Nutrition Program (NNP), particularly NNP II, the National School Feeding Strategy (NSFS), and the Seqota Declaration to enhance adolescent nutrition [18]. However, undernutrition among adolescent girls persists, with various location-specific factors contributing to the issue.

Compared to under-five children and maternal nutrition, adolescent nutrition receives less attention, hindering efforts to break the cycle of nutritional problems across generations. Evaluating the current nutritional status of adolescent girls is crucial for designing and implementing intervention programs. However, there is limited knowledge about the nutritional status of adolescent girls in the study area. However, there is limited knowledge about the nutritional status of adolescent girls in the study area. Therefore, this study aimed to identify the undernutrition status of adolescent girls and their associated factors in Diga District, East Wollega Zone, Ethiopia.

## Methods

### Study area and period

The study was conducted in Diga district, East Wollega Zone, Ethiopia, from July 10^th^ to August 10^th,^ 2023. Diga District is located about 341 kilometers to the west of Addis Ababa, the capital city of Ethiopia, and around 12 kilometers from Nekemte town, in the west direction. The primary economic activity of the district’s residents centered on the agricultural products. Diga District is divided into 24 kebeles and has four health centers and 23 health posts to address the health needs of the community. As per the 2007 national census report, the district’s total population was 66,689, comprising 33,896 men and 32,793 women, residing in 21,018 households. The total number of adolescents in the District is 20909, whereas the number of adolescent girls is 11234.

### Study design and population

A community-based cross-sectional study design was utilized. The source population consisted of all households with adolescent girls aged between 10 and 19 years in the Diga District, while the study population comprised all households with adolescent girls in the selected kebeles in the Diga District during the data collection period. The study included adolescent girls between 10 and 19 years residing in the selected kebeles.

### Sample size determination

The sample size was determined using the formula for estimating the difference between two population proportions, considering a 95% confidence interval, 80% power, and a 1:1 ratio of unexposed to exposed individuals, using Epi Info software version 7. The adjusted odds ratio for weekly meat consumption (one to two times) in the Arsi Zone, Aseko District (AOR = 2.52) [29] was applied to estimate the sample size of this study. After including a 10% adjustment for the non-respondent rates and design effects, the total sample size was set at 651.

### Sampling procedure

The first eight kebeles were randomly selected from a total of 24 kebeles in the Diga district. Families with at least one adolescent girl were identified from the family folder reports obtained from health extension workers. The study participants were allocated to each of the selected kebeles based on the proportion of total households with at least one adolescent girl. Systematic sampling was employed to select households using a list of households with at least one adolescent girl as a sample frame, with adolescents serving as the sampling unit.

**Fig 1:**
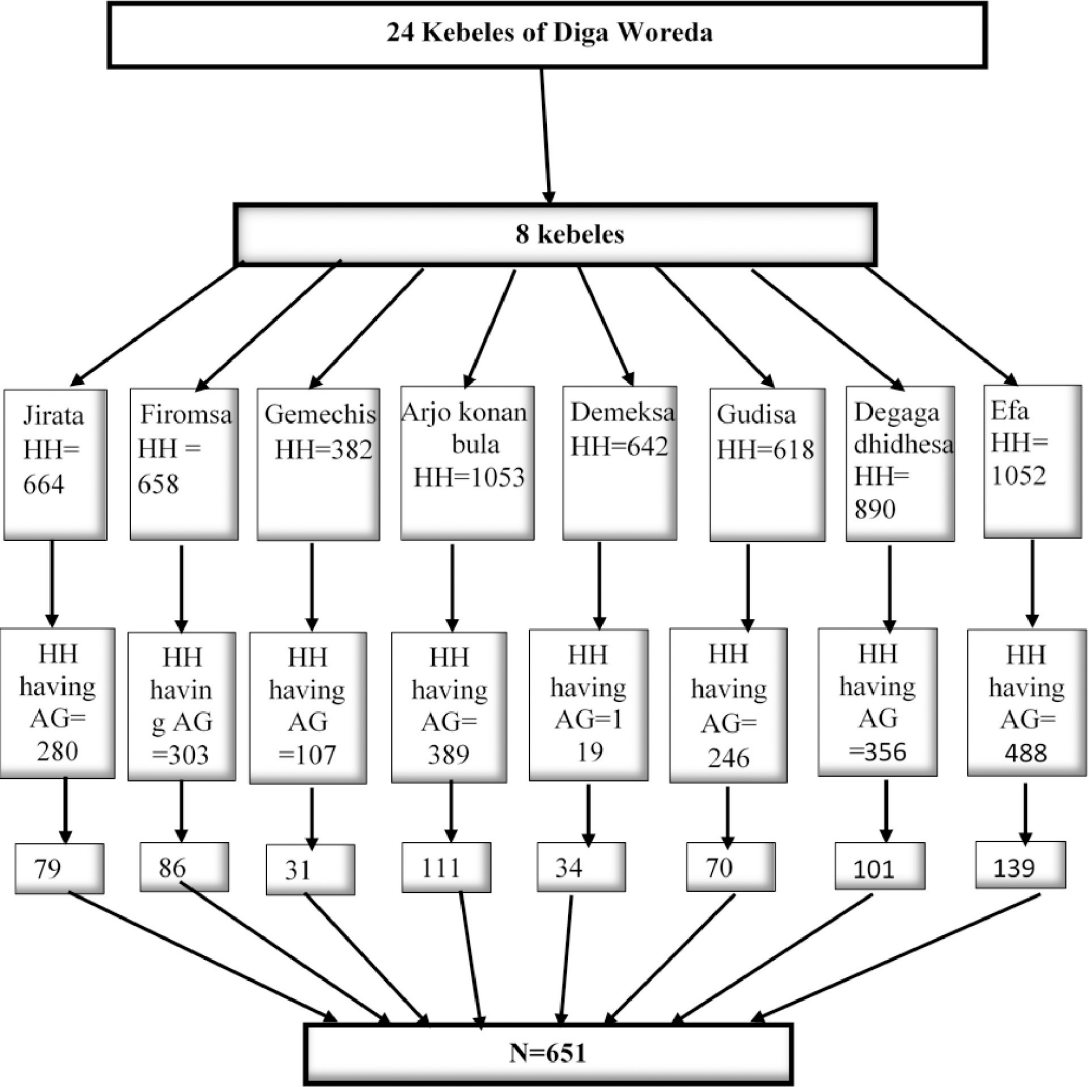
Summary of the sampling procedure for the study in Diga District, East Wollega Zone, 2023. Keys: HH: Household; AG = Adolescent girls; SRS = Systematic Random Sampling and N = Total sample size.

Moreover, unique consecutive numbers were reassigned to establish a sampling frame for eligible households with adolescent girls. The study participants were chosen using a systematic sampling technique with a sampling interval (K) determined from the total number of eligible households, where K^th^ = 2288/651 = 3. Subsequently, through a lottery method, random starts were selected, and every 3rd adolescent girl was chosen from the family folder until reaching the required sample size [Fig 1].

### Operational definitions

- **Undernutrition**: is the presence of stunting and/or thinness (<˗2 standard deviation) [30].
- **Thinness**: is the proportion of adolescent girls with a value body mass index for age Z–score < -2 standard deviation [30].
- **Stunting:** is the proportion of adolescent girls with value height-for-age Z-score <-2 standard deviation [30].
- **Individual Dietary Diversity Score**: the sum of food groups eaten by adolescent girls over the last 24 hours [31].
- **Poor dietary diversity**: Adolescent girls who consume less than five food groups from the ten food items in the previous 24 hours [32].
- **Good dietary diversity**: Adolescent girls who consumed (≥5 food groups) from the ten food items in the previous 24-hours [32].
- **Food security**: Based on the Household Food Insecurity Access Scale (HFIAS), a score (0–27) of 0–1 is food security [33].
- **Food insecurity:** Based on HFIAS, score (0–27) of 2 and above is food insecurity [33].

### Data collection tool and procedure

Structured interviewer-administered questionnaires were utilized to gather data, adapted from various literature reviews and previous similar studies [21, 28, 29, 34, 35], along with anthropometric measurements of adolescent girls meeting the study’s inclusion criteria. The questionnaire encompassed information on socio-demographic factors, household income, household environment, behavioral factors, dietary habits, health status, and agricultural production-related aspects [S1 File].

A qualitative recall method was employed to record all foods consumed by each adolescent female in the preceding 24 hours to evaluate their dietary consumption pattern. The individual dietary diversity score (IDDS), with the mean Dietary Diversity Score (DDS), is estimated based on these records [31]. Additionally, food frequency patterns were obtained from adolescent girls by inquiring about the food categories they consumed most frequently in the month leading up to the data collection.

The level of household food security was assessed using the Household Food Security Access Scale (HHFAS), a structured, standardized, and validated tool primarily developed by FANTA version 3 to determine the food security status of households [36].

### Anthropometric measurement

The height of the adolescent girls were measured using a portable hardwood height-measuring board with a sliding head bar, while their weight was recorded using portable electronic digital scales, using the WHO recommendations [37]. They were instructed to stand upright with shoulders level, hands at their sides, and heels together comfortably. The girls stood on the footplate of the stadiometer without shoes, positioned with their heels close together, legs straight, arms at their sides, and shoulders relaxed. Buttocks, scapulae, calf, heel, and back of the head touch with the vertical backboard equipped with a sliding head bar. The study participants were asked to inhale and stand fully erect without altering the position of their heels. We removed hair ornaments or buns to ensure an accurate measurement. The perpendicular headpiece of the stadiometer was gently lowered to touch the crown of the adolescent’s head. Then, height was measured to the nearest 0.1 centimeters (cm).

Similarly, adolescent girls were requested to take off their shoes and wear light clothing before being weighed using a calibrated portable digital scale. Privacy was maintained privacy by allowing them to wear a light cloth or providing them gown. The weight was recorded to the nearest 0.1 kilogram (kg) through repeated measurements, ensuring accuracy and consistency by using specifically trained measurers. If the difference between the two measurements exceeds 1cm (100g for weight), the measurement was repeated, and the closest two measures were averaged. Additionally, the calibration of the scale was regularly verified by placing standard 2kg iron bars on the scales and adjusting the calibration as needed to maintain accuracy.

### Data quality control

To ensure data quality, several measures were implemented in the study. Initially, the structured questionnaire was prepared in English, then translated into the respondents’ native language (Afaan Oromo) [S2 File] and back-translated into English to ensure comprehension and consistency. Data collectors and supervisors underwent a one-day training session covering the study’s objectives, sampling techniques, ethical considerations, and data collection methods to standardize procedures. A pretest was conducted on 5% of the study sample in the Wayu Tuka district before actual data collection to enhance instrument clarity and familiarize data collectors with the tools.

We calculated Technical Errors of Measurement (TEM) during training to reduce intra- and inter-observer variability. Data collectors with TEM below 2% were selected for data collection to maintain data quality and minimize measurement errors. Digital weight scales were checked for accuracy before each weight measurement to ensure precise readings. Two health officers supervised the data collection process to ensure completeness, validity, and consistency of the collected data, with oversight from the primary investigator. After data collection, questionnaires were reviewed for completeness and consistency before data entry.

### Data processing and analysis

Data were coded on a coding sheet to reduce errors, and Epi-Data 4.6 software was used for data entry. The data was exported to SPSS version 25 for statistical analysis. The WHO Anthro Plus version 1.0.4 software converted nutritional data (age, height, weight) into Z-scores. The Body Mass Index for Age Z-Score (BAZ) and Height for Age Z- Z-Score (HAZ) using WHO 2007 population references. The composite variables (HAZ and BAZ) WHO Z-scores are obtained from their respective anthropometric measurements using AnthroPlus software. A score of <-2 SD of BMI-for-age and height-for-age was coded as ’1’ and classified as thin and stunted, respectively. On the other hand, a score of >-2 SD of BMI-for-age and height-for-age was coded as ’0’ and classified as not thin and stunted, respectively.

Descriptive statistics such as frequencies, proportions, and correlations were used to present the study findings. The Kolmogorov–Smirnov test at a p-value greater than 0.05 was used to check all continuous variables for normality. Before including independent variables, multicollinearity was checked using a Variance Inflation Factor (VIF) <10 and Tolerance greater than 0.1. The Hosmer and Lemeshow goodness of fit test was used to assess the fitness of the model during the multivariable analysis, and the model was found to be adequate (>60%).

Bivariable and multivariable logistic regression was conducted to assess the association between the dependent variables (stunting and thinness) and the independent variables. A variable with a p-value of less than 0.25 was considered for the multivariable logistic regression analysis. The strength of association was measured using both crude and adjusted odds ratios along with 95% confidence intervals. A p-value less than 0.05 was considered to declare the statistical significance of the dependent variable with the independent variable.

### Ethical considerations

Before starting the study, we addressed various ethical considerations. We obtained ethical clearance from the Wollega University Institutions of Health Sciences’ Ethical Review Committee. The clearance, granted on May 29, 2023, with Reference No. WU/RD/669/2023 was officially approved. Approval was also obtained from the Diga District Health Office and the administration offices in the study area. The research objectives and purpose were explained to the study participants before the actual collection of data. Written informed consent was obtained from adolescent girls aged 18 years or above. Informed verbal and written consent was obtained from the parents/caregivers of adolescent girls aged less than 18 years. We ensured the confidentiality of information, and data collection was carried out confidentially and securely.

## Results

### Socio-demographic characteristics

In this study 627 out of the 651 invited teenage girls participated, resulting in a response rate of 96.3%. The average age of the participants was 14.48 years with a standard deviation of ± 2.57 (14.48± 2.57) (range 10–19 years). The majority of the respondents, 538 (85.8%), mentioned that they lived with both parents. Additionally, 549 (87.6%) of the teenage girls were students. The study also revealed that 229 (36.5%) of the fathers of the girls had completed primary school. Regarding occupation, 423 (67.5%) of the fathers were involved in farming. Among the mothers of the respondents, 322 (51.4%) had completed primary education, and 418 (66.7%) were engaged in farming. The study also explored the size of the participants’ families, showing that 556 (88.7%) came from families with more than five members. In terms of monthly income, 330 (52.6%) of the families reported a monthly income between 1000 and 2000 Ethiopian Birr [Table 1].

**Table 1:** Socio-demographic characteristics of family and adolescent girls Diga District, East Wollega Zone, Ethiopia 2023 (N = 627).

Variable	Category	Frequency	Percentage (%)
Age of adolescent girls (years)	10–14	316	50.4
(14.48± 2.57)	15–19	311	49.6
Adolescent currently living with	Primary family (father and mother)	538	85.8
	Mother	45	7.2
	Relatives/other	44	7.0
The current job of an adolescent girl	Student	549	87.6
	Private work/business	41	6.5
	Maid	37	5.9
Parents’ marital status	Married	557	88.8
	Divorced	20	3.2
	Widowed	50	8.0
Household head	Male	554	88.4
	Female	73	11.6
Father’s level of education	No formal education	51	8.1
	Primary education (Grade 1–8)	229	36.5
	Secondary education (Grade 9–12)	183	29.2
	College and above	164	26.2
Mother’s level of education	No formal education	133	21.2
	Primary education (Grade 1–8)	322	51.4
	Secondary education(Grade 9–12)	138	22.0
	College and above	34	5.4
Father’s occupation	Farmer	423	67.5
	Merchant	37	5.9
	Daily laborer	17	2.7
	Government employer	150	23.9
Mother’s occupation	Farmer	418	66.7
	Housewife	89	14.2
	Merchant	69	11.0
	Daily laborer	16	2.5
	Government employer	35	5.6
Family size	< 5	71	11.3
	≥ 5	556	88.7
Family’s monthly income (ETB)	< 1000 Ethiopia	33	5.3
	1000–2000 Ethiopia	330	52.6
	2001–3000 Ethiopia	58	9.3
	>3000 Ethiopia	206	32.8

Keys: ETB: Ethiopian Birr

### Health-related characteristics of adolescent girls

Among the teenagers involved in the study, 396 (63.2%) had already experienced menstruation, and among those who experienced it, 385 (97.2%) had their first period at the age of 14 years or younger. The study also indicated that a significant number of participants, specifically 474 (75.6%), mentioned that they had no access to health and nutrition information [Table 2].

**Table 2:** Health-related characteristics of adolescent girls Diga District, East Wollega Zone, Ethiopia, 2023(n = 627).

Variables	Category	Frequency	Percentage (%)
Adolescent menstruation status	Started	396	63.2
	Not started	231	36.8
Age at menarche	≤14	385	97.2
	>14	11	2.8
Access to health and nutrition information	Yes	153	24.4
	No	474	75.6
Source of health and nutrition information	Health profession/HEW	64	41.8
	Mass media	35	22.9
	Family	21	13.7
	From school	33	21.6
History of illness in the last two weeks	Yes	35	5.6
	No	592	94.4

### Dietary diversity score of adolescent girls

**Fig 2:**
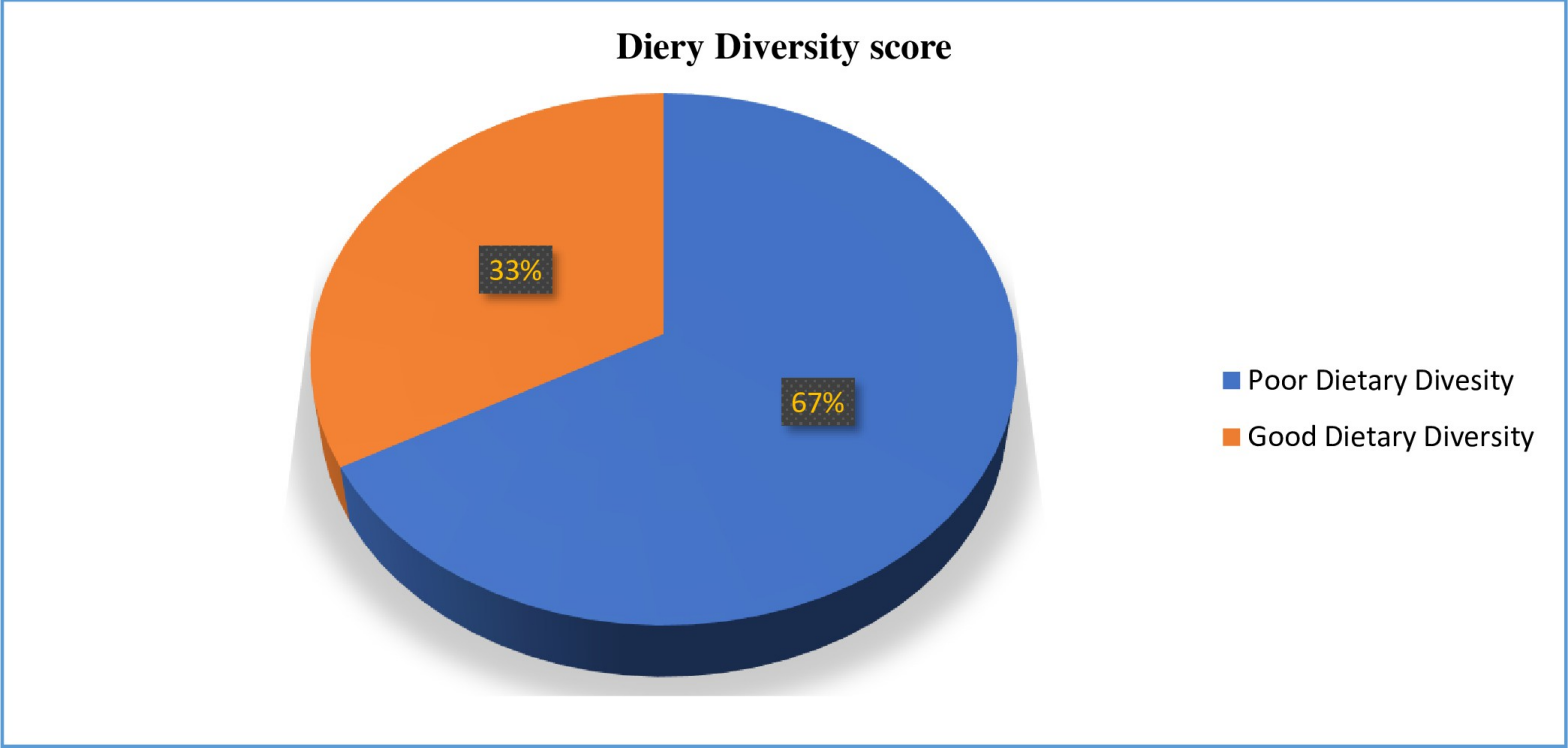
Dietary diversity score of adolescent girls in Diga District, East Wollega Zone, Ethiopia, 2023 (N = 627).

The dietary diversity scores collected in the study ranged from a minimum of 1 to a maximum of 9, with an average (SD) score of 4.06 (1.21). A notable portion of the participants, around 420 (67%), had a dietary diversity score of less than five food groups [Fig 2].

### Food frequency pattern of adolescent girls

During the one-month duration of the study, it was observed that a large majority of teenage girls, specifically 437 (69.7%), consumed cereal and grain items 2–3 times daily. On the other hand, meat was reported to be the least frequently consumed food item among teenage girls, and no one reported consuming fish products within one month [Table 3].

**Table 3:** Food items and frequencies consumed by adolescent girls in the last month in Diga District, East Wollega Zone, Ethiopia, 2023.

Food items	Frequencies of the food items consumed over the last month								
	>3times/day	2–3 times/day	Once/day	3–4 times/week	2 times/week	One time/week	2–3 times/month	Once /month	Never
Starch staple foods (cereals)	41 (6.5%)	437 (69.7%)	149 (23.8%)	0 (0%)	0 (0%)	0 (0%)	0 (0%)	0 (0%)	0 (0%)
Vegetables^a^	0 (0%)	8 (1.3%)	25 (4.0%)	52 (8.3%)	204 (32.5%)	324 (51.7%)	14 (2.2%)	0 (0%)	0 (0%)
Tubers and roots	0 (0%)	0 (0%)	81 (12.9%)	502 (80.1%)	37 (5.9%)	7 (1.1%)	0 (0%)	0 (0%)	0 (0%)
Any fruit^b^	0 (0%)	0 (0%)	26 (4.1%)	10 (1.6%)	111 (17.7%)	400 (63.8%)	80 (12.8%)	0 (0%)	0 (0%)
Meats	0 (0%)	0 (0%)	0 (0%)	0 (0%)	0 (0%)	0 (0%)	0 (0%)	229 (36.5%)	398 (63.5%)
Eggs	0 (0%)	0 (0%)	0 (0%)	25 (4.0%)	58 (9.2%)	200 (31.9%)	150 (23.9%)	20 (3.2%)	174 (27.8%)
Fish	0 (0%)	0 (0%)	0 (0%)	0 (0%)	0 (0%)	0 (0%)	0 (0%)	0 (0%)	627 (100%)
Beans, Lentils, peas	0 (0%)	51 (8.1%)	214 (34.1%)	300 (47.9%)	62 (9.9%)	0 (0%)	0 (0%)	0 (0%)	0 (0%)
Milk products	0 (0%)	0 (0%)	75 (12.0%)	29 (4.6%)	102 (16.3%)	209 (33.3%)	58 (9.3%)	78 (12.4%)	76 (12.1%)
Nuts & seeds	0 (0%)	0 (0%)	113 (18.0%)	67 (10.7%)	112 (17.9%)	335 (53.4%)	0 (0%)	0 (0%)	0 (0%)
Tea/coffee	0 (0%)	123 (19.6%)	491 (78.3%)	0 (0%)	0 (0%)	0 (0%)	0 (0%)	0 (0%)	13 (2.1%)

Keys: a: dark-green leafy vegetables; b: fruits such as mango, avocado, banana, etc.

### Household Food Security Access Scale condition (HHFS)

**Fig 3:**
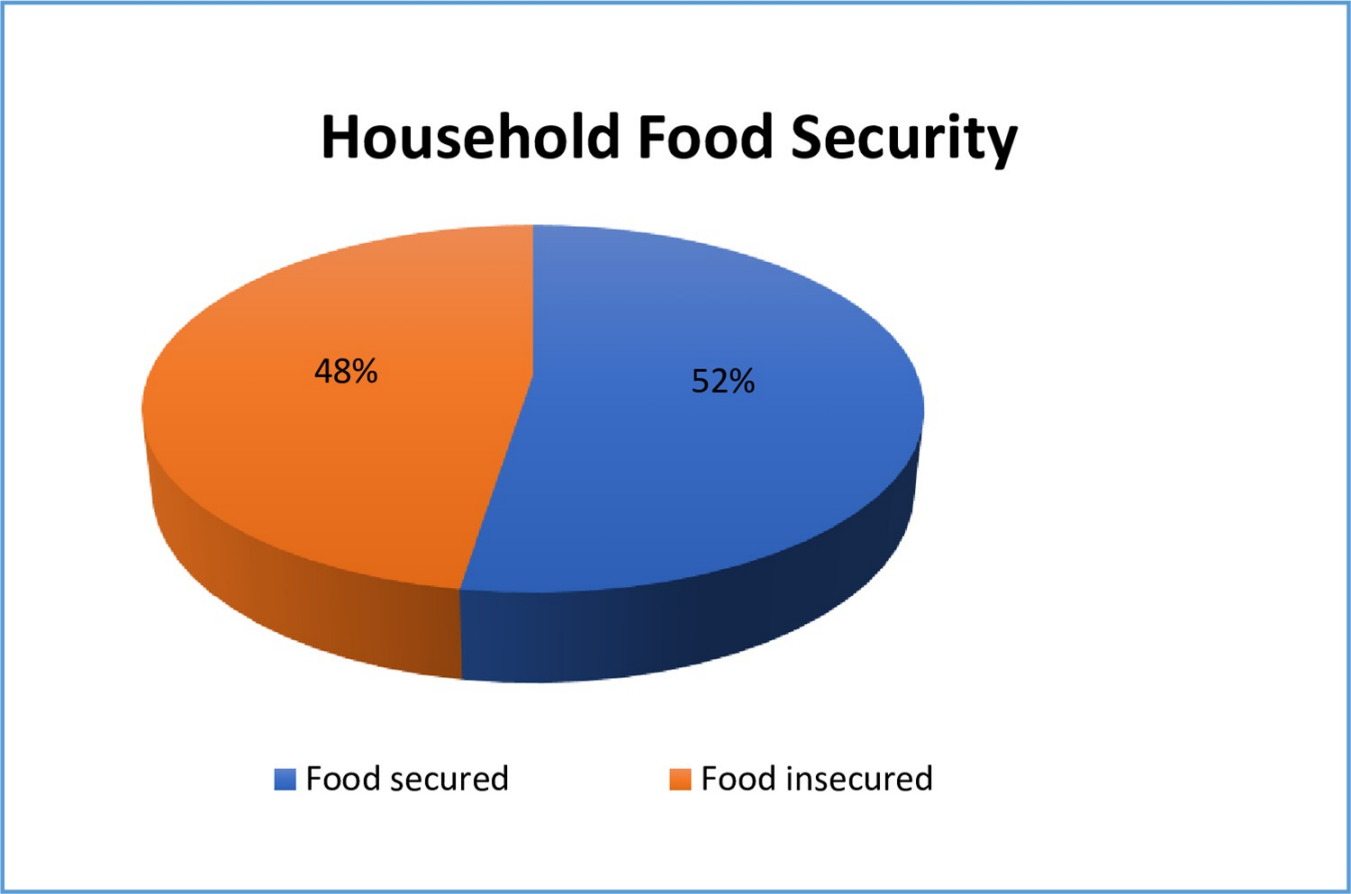
Household food security of adolescent girls in Diga District, East Wollega Zone, Ethiopia, 2023 (N = 627).

The information regarding dietary habits and food security was gathered from a household member responsible for food preparation in each participant’s household. The study displayed that more than half of the families of adolescent girls, specifically (52.5%), were classified as food insecure [Fig 3].

### Household environmental and agricultural production-related factors

As per the study results, 551 (87.9%) of the participants mentioned using drinking water from improved sources. Additionally, concerning the presence of latrines, the study revealed that 614 (97.9%) of the participants had a latrine within their premises. Following the study, 315 (50.2%) of the respondents reported having a home garden. Out of the total number of participants (627), 573 (91.4%) individuals stated they had fertile agricultural land. Among all the respondents, 308 individuals (53.8%) reported cultivating a variety of crops [Table 4].

**Table 4:** Households’ environmental and agricultural production related characteristics of adolescent girls in Diga District, East Wollega Zone, Ethiopia, 2023(N = 627).

Variables	Category	Frequency	Percentage (%)
Source of drinking water	Tap water	400	63.8
	Protected spring water	151	24.1
	Unprotected spring water	76	12.1
Has water treatment method	Yes	400	63.8
	No	227	36.2
Has latrine	Yes	614	97.9
	No	13	2.1
Handwashing after toilet	Yes	511	81.5
	No	116	18.5
Cleansing agent use	Water and soap	488	95.5
	Water only	23	4.5
Home Gardening	Yes	315	50.2
	No	312	49.8
Purpose of home gardening	For sale	8	87.3
	For consumption	275	2.5
	For both	32	10.2
Having fertile agricultural land	Yes	573	91.4
	No	54	8.6
Ownership of agricultural land	Own	484	84.5
	Rent	89	15.5
Size of agricultural land	≤ 2 hectares	449	78.4
	>2 hectares	124	21.6
Annual agricultural production (including all items) adequate for consumption	Yes	261	45.5
	No	312	54.5
Produce varieties of crops	Yes	308	53.8
	No	265	46.2
Purpose of Variety of Crop Production	For sale	20	6.5
	For consumption	34	11.0
	For both	254	82.5
Poultry or other animals	Yes	467	74.5
	No	160	25.5
Produce Variety of crops	Yes	308	53.8
	No	265	46.2

### Prevalence of undernutrition among adolescent girls

**Fig 4:**
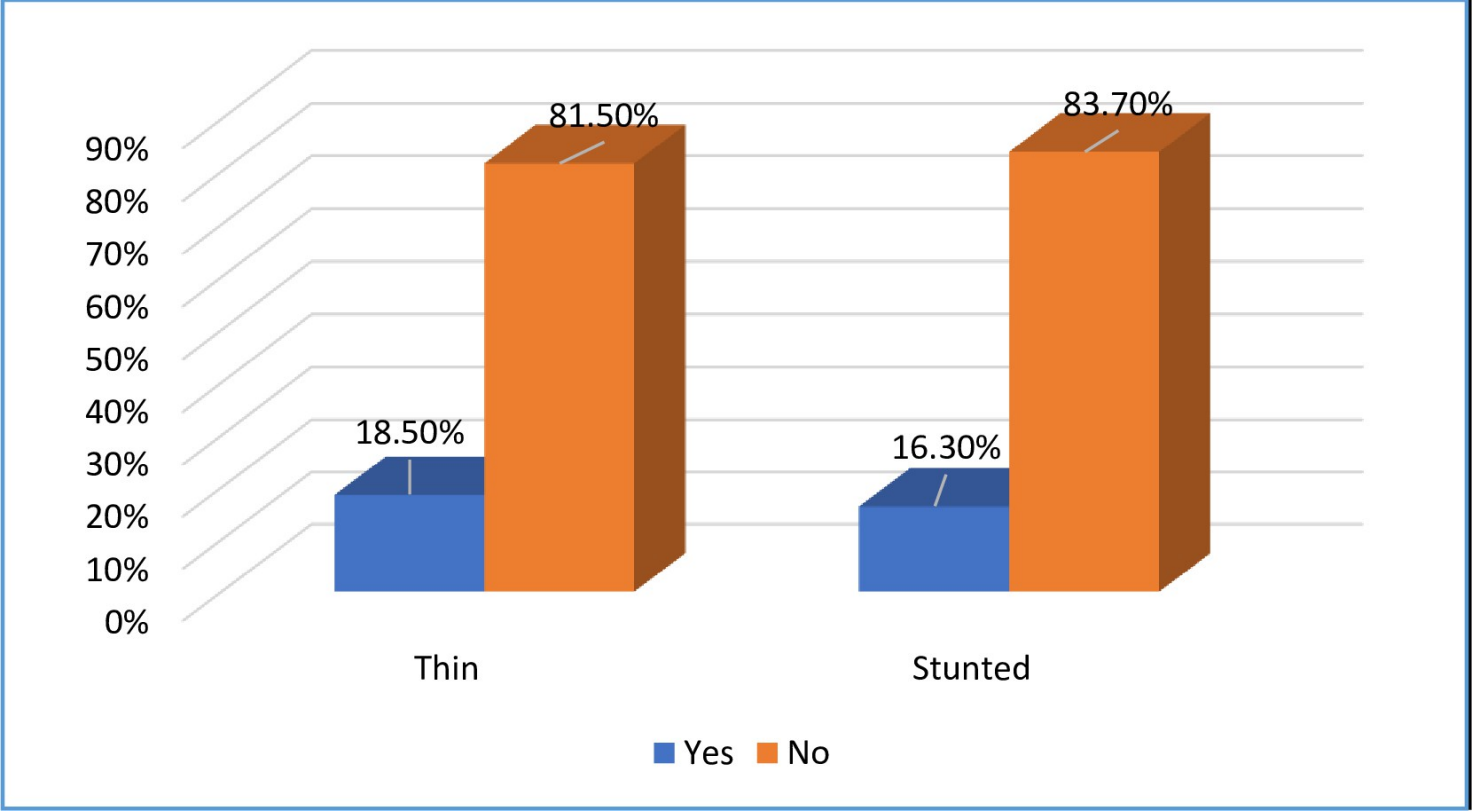
Prevalence of undernutrition among adolescent girl in Diga District, East Wollega Zone, Ethiopia, 2023 (N = 627).

Undernutrition among adolescent girls was identified through stunting and thinness. The study found that out of the 627 study participants, around 116 individuals, accounting for 18.5% of the sample, were classified as thin with a 95% CI (15.4–21.5). Additionally, the study noted that 102 participants, representing 16.3% of the sample, were classified as stunted with a 95% CI (13.5–19.3) [Fig 4].

### Factors associated with stunting in adolescent girls

Firstly, a bivariable analysis is conducted on independent variables associated with stunting with a p-value < 0.25. Nine independent variables: mother’s occupation, family size, current job status of the adolescent girls, access to health and nutritional information, water treatment method, varieties of crop production, age of the adolescents, dietary diversity scores, and household’s food security.

Secondly, we entered these nine variables into a multivariable logistic to identify the independent variables significantly associated with stunting. After the analysis, three variables (access to health and nutritional information, variety of crop production, and household food insecurity) were found to be significantly associated with stunting with a p-value < 0.05.

The analysis showed that adolescent girls who did not have access to health and nutritional information were found to have 3.36 times higher odds of being stunted compared to those who had access (AOR: 3.36; 95% CI: 1.37–8.23). Furthermore, adolescent girls whose households did not produce a variety of crops had 1.66 times higher odds of developing stunting compared to their counterparts (AOR: 1.66; 95% CI: 1.03–2.65). Similarly, the odds of being stunted were 2.76 times higher among adolescents from households experiencing food insecurity compared to their counterparts (AOR: 2.76; 95% CI: 1.49–5.11) [Table 5].

**Table 5:** Factors associated with stunting among adolescent girls in Diga District, East Wollega Zone, Ethiopia, 2023(N = 627).

Variables	Category	Stunted		COR(95%CI)	AOR (95%)	P value
		Yes	No			
Mother’s occupation	Farmer	66	352	1.33(0.56–3.19)	1.79(0.62–5.20)	0.28
	Housewife	15	74	1.23(0.46–3.34)	1.86(0.56–6.16)	0.31
	Merchant	8	61	1.91(0.63–5.78)	3.18(0.80–12.61)	0.1
	Daily laborer	6	10	0.42(0.11–1.54)	0.75(0.17–3.45)	0.72
	Governmental employer	7	28	1	1	1
Family size	< 5	8(11.3%)	63(88.7%)	1	1	
	> = 5	94(16.9%)	462(83.1%)	1.60(0.74–3.46)	1.49(10.52–4.27)	0.46
The current job of an adolescent girl	Student	86(15.7%)	463(84.3%)	1	1	
	Private work/business	6(16.2%)	31(83.8%)	1.74(0.821–3.673)	0.92(0.35–2.42)	0.86
	Maid	6(16.2%)	31(83.8%)	1.04(0.42–2.57)	0.85(0.23–3.08)	0.80
Access to health and nutritional information	Yes	13(8.5%)	140(61.5%)	1	1	
	No	89(18.8%)	385(81.2%)	2.49(1.35–4.59)	**3.36(1.38–8.23**)	**0.01** *
Water treatment method	Yes	58(14.5%)	342(85.5%)	1	1	
	No	44(19.4%)	183(80.6%)	1.42(0.92–2.18)	1.29(0.73–2.28)	0.38
Variety of crop production	Yes	38(12.3%)	270(87.7%)	1	**1**	
	No	57(27.1%)	208(72.9%)	1.95(1.56–4.54)	**1.66(1.03–2.65)**	**0.04** *
Age of adolescent	10–14 years	44(13.9%)	272(86.1%)	0.71(0.46–1.08)	1.07(0.58–1.97)	0.82
	15–19 years	58(18.6%)	253(81.4%)	1	1	
DDS	Poor dietary diversity score (< 5)	86(20.5%)	334(79.5%)	3.07(1.75–5.39)	1.87(0.98–3.57)	0.06
	Good dietary diversity score (≥5)	16(7.7%)	191(92.3%)	1	1	
HHFS	Secured	25(7.6%)	304(92.4%)	1	1	
	Insecure	77(25.8%)	221(74.2%)	4.24(2.61–6.87)	**2.76(1.49–5.11)**	**0.01** *

Keys; DDS: Dietary Diversity Score, HHFS: Household’s Food Security

*: Significant at p-value <0.05 in multivariable logistic regression

### Factors associated with thinness in adolescent girls

In the study, a bivariable analysis was conducted to identify potential factors associated with thinness with a p-value < 0.25. Based on this criterion, eight variables: educational status of the father, occupational status of the mother, adolescent currently living with whom, age of first menstruation, wash your hands after toilet, fertile agricultural land, dietary diversity score, and household food security were identified as candidate variables for multivariable logistic analysis.

After the multivariable logistic regression analysis, dietary diversity score and household food insecurity were found to be significantly associated with thinness with a p-value of < 0.05. Accordingly, adolescent girls with a poor dietary diversity score were 7.52 times more likely to be thin compared to those with a good dietary diversity score (AOR: 7.52; 95% CI: 2.92–19.39). Furthermore, adolescent girls from food-insecure households were 3.69 times more likely to be thin compared to those from food-secured households (AOR: 3.69; 95% CI: 1.96–6.33) [Table 6].

**Table 6:** Factors associated with thinness among adolescent girls in Diga District, East Wollega Zone, Ethiopia, 2023(N = 627).

Variables	Category	Thinness		COR (95%CI)	AOR(%CI)	p-value
		Yes	No			
Father’s education level	No formal education	9(20.5%)	35(79.5%)	1.31(0.56–3.03)	1.73(0.48–6.19)	0.40
	Primary education(Grade 1–8)	35(14.8%)	201(85.2%)	0.88(0.51–1.53)	1.19(0.42–6.19)	0.73
	Secondary education(Grade9-12)	45(24.6%)	138(75.4%)	1.66(0.97–2.82)	2.09(0.81–5.38)	0.13
	College and above	27(16.5%)	137(83.5%)	1	1	
Mother’s occupation	Farmer	65	353	1.36(0.57–3.24)	1.42(0.32–6.24)	0.64
	Housewife	25	64	0.64(0.25–1.65)	0.40(0.09–1.91)	0.25
	Merchant	12	57	1.19(0.42–3.35)	0.38(0.07–2.12)	0.27
	Daily laborer	7	9	0.32(0.09–1.67)	1.02(0.10–10.16)	0.98
	Government employer	7	28	1	1	
Adolescent currently living with	Primary family(father and mother)	94(17.3%)	450(82.7%)	1	1	
	Mother only	8(19.1%)	34(80.9%)	1.13(0.51–2.51)	0.79(0.31–2.08)	0.65
	Relatives	14(34.1%)	27(65.9%)	2.48(1.25–4.91)	2.03(0.84–4.89)	0.12
Menarche age	<14	60(15.7%)	323(84.3%)	0.22(0.07–0.67)	0.29(0.07–1.28)	0.10
	≥14	6(46.2%)	7(53.8%)	1	1	
Wash your hands after toilet frequently	Yes	87(17.0%)	424(83.0%)	1	1	
	No	29(25.0%)	87(75.0%)	1.63(1.01–2.62)	1.65(0.81–3.36)	0.17
Presence of fertile agricultural land	Yes	52(21.6%)	189(78.4%)	1	1	
	No	64(16.6%)	322(83.4%)	0.72(0.48–1.09)	1.06(0.39–2.91)	0.91
DDS	Poor dietary diversity score (<5)	105(25.0%)	315(75.0%)	5.94(3.11–11.33)	**7.52(2.92–19.39)**	**0.01**
	Good dietary diversity score (≥5)	11(5.3%)	196(94.7%)	1	1	
HHFS	Secured	29(8.8%)	300(91.2%)	1	1	
	Insecure	87(29.2%)	211(70.8%)	4.27(2.70–6.73)	**3.69(1.96–6.93)**	**0.01**

Keys: DDS; Dietary Diversity Score, HHFS; Household’s Food Security

*: Significant at p-value <0.05 in multivariable logistic regression

## Discussion

This study aimed to determine the prevalence of undernutrition and associated factors among adolescent girls in the Diga district, Ethiopia. It provides insights into undernutrition and associated factors among adolescent girls in Diga District. The study found that the prevalence of stunting was 16.3% and thinness was 18.5%. Stunting is significantly associated with a lack of access to health and nutrition information, households producing less variety of crops, and food insecurity within the household. Likewise, poor dietary diversity scores and household food insecurity are independently associated with thinness.

Comparing the prevalence of stunting with previous studies, the rates in Diga district was similar to those in Aseko (20.2%), Debark (20.1%), and Abuna Gindeberet (15.4%) districts in Ethiopia [18, 20, 25, 29] but lower than those in the Hawzen (33.2%) and Damot Sore (29.6%) districts, in Ethiopia [19, 38]. The current study primarily focused on the rural while the study done in Damot Sore included girls from rural and urban [38]. Previous study also revealed that undernutrition among adolescent girls is more common in rural than urban areas [11]. Furthermore, the study done in Hawzen district is a school-based study that included girls attending their academy [19]. Conversely, the stunting rate in Diga was higher than the findings in the Mirab-Armachiho district (10.3%) Southern Ethiopia (8.8%) [23, 24], and Southeast Asia (11.1%) [9]. The study settings, socioeconomic status, feeding culture, and access to nutritional information are probably the reasons for the variations reported [18, 19, 38].

In the same manner, the prevalence of thinness in various districts of Ethiopia is consistent with previous findings. In Babile, Aseko, Damot Sore, Mirab-Armachiho, and Abuna Gindeberet districts in Ethiopia, ranging from 14.2% to 19.5% [24, 25, 29, 38, 39]. In contrast, the rate of thinness is lower than the findings in South Ethiopia (27.5%) and Hawzen districts in Ethiopia [19, 23], as well as, in Bangladesh, India, and Nepal [26, 40, 41]. The differences in stunting and thinness prevalence across these studies could be attributed to variations in socioeconomic, seasons, feeding practice, dietary, environmental factors (seasonal variation and political instability), and health and nutrition access to information-related factors [18, 19, 23, 38].

In this study, adolescents who lacked access to health and nutrition information were over three times more likely to be stunted compared to adolescent girls who had access to such information. This finding is consistent with a study conducted in the five districts in Amhara Region, Ethiopia [27]. Adolescents have better nutrition information, know their dietary choices, and have better feeding practices to maintain their nutritional status [42]. Educating and extending adolescent girls about health and nutrition is important.

According to the current study, stunting is more likely to occur in adolescent girls from households not produce a variety of crops. This finding is in harmony with the previous studies [43–45]. Adolescent girls who feed on a diverse crops can obtain essential nutrients that improve their growth and development. Promoting crop diversity improves the nutritional status of adolescent girls, as well as benefits their overall health and contributes to food security. Additionally, households that produce a variety of crops likely have sufficient crop production to provide better quality and frequency of meals for their families. In Ethiopia, families who produce a diverse range of crops tend to have better incomes. Similarly, the present study displayed that adolescent girls from food-insecure households were more than twice as likely to be stunted compared to those from food-secure households. This finding is in harmony with the previous studies’ findings [18, 22, 24, 46]. The prevalence of chronic food insecurity can lead to stunting, which is a crucial component in the long-term nutritional issues faced by adolescent girls. In households with food insecurity, access to a diverse array of nutrient-rich foods is often limited, leading to alterations in physical and physiological development.

The finding also indicated that adolescent girls with poor dietary diversity scores were more likely to be thinner compared to their counterparts. This is harmonious with the findings of districts in different districts in Ethiopia and other countries [24, 25, 27]. Adolescence is a period of acceleration with significant increases in weight, height, and muscle mass [47]. This rapid growth and development requires higher energy and nutrient intake to support the body’s changing needs. Also, the onset of menstruation during adolescence requires replacing the blood and nutrients lost during the menstrual cycle.

In parallel, this study showed that the odds of thinness are higher among adolescent girls with food insecurity. This result is consistent with a study conducted in the Mirab-Armachiho district in Ethiopia and Nepal [24, 41]. A systematic review and meta-analysis done in Ethiopia also shed similar findings including both male and female adolescents [20]. Those who are food insecure are more vulnerable to thinness because food is essential for adolescent physical growth in addition to its health benefits. Since food insecurity restricts access to nutritious foods, lack of access to diverse and nutritious food options can lead to undernutrition, despite having the right information.

### Study limitations

This study excluded urban adolescent girls focusing on the rural girls. This could affect the generalizability of the findings for all adolescent girls. Recall bias may be present as the Dietary Diversity Score (DDS) was calculated based on 24-hour food consumption and food frequency questions. Therefore, researchers are recommended to consider the limitations of this study for utilizing it.

## Conclusion

This study revealed a low level of stunting and thinness among the adolescent girls in the Diga district. Lack of access to health and nutrition information, limited crop diversity in households, and household food insecurity were strongly associated with stunting. Furthermore, poor dietary diversity and household food insecurity were significantly linked to thinness among respondents. These findings highlight the importance of addressing these factors to enhance the nutritional status of adolescent girls and alleviate the burden of undernutrition in the population. Interventions that focus on improving household-level crop diversity, enhancing food security, providing adequate health and nutrition information, and encouraging income-generating activities for adolescent girls can help improve their access to nutritious foods and healthcare, contributing to better nutritional outcomes.

## Supporting information

S1 File English version of the questionnaire. (DOCX)

S2 File Afan Oromo version of the questionnaire. (DOCX)

S1 Dataset (XLSX)

## Abbreviations and acronyms

AOR: Adjusted Odds Ratio
BMI: Body Mass Index
BAZ: Body Mass Index for Age Z-Score
CI: Confidence Interval
HAZ: Height for Age Z-Score
SD: Standard Deviation
SPSS: Statistical Package of Social Science
HFSAS: Household Food Security Asses Scale
