# Supplementary material for: Undernutrition and associated factors among adolescent girls in Diga District, East Wollega Zone, Ethiopia
Source: PLoS One. 2024 Oct 29;19(10):e0310225. doi: 10.1371/journal.pone.0310225 (PMC11521244; doi:10.1371/journal.pone.0310225)
Supplement: S1 File — (DOCX) [file pone.0310225.s001.docx]

## **English Version of the Questionnaire**

**Undernutrition and Associated factors among adolescent girls in Diga district East Wallaga, Ethiopia**

**Consent form that certifies the respondent’s agreement before the interview**

**Introduction**

My name is ________________________I am working as a data collector for the study being conducted to assess undernutrition and associated factors among adolescents’ girls in Diga District, East Wallaga, Ethiopia. The investigation was carried out using a structured interviewer-administered questionnaire and anthropometric measurements. Respondent names and other personal identifiers will not be recorded on the data collecting forms, and the information you provide us will be kept confidential and used only for the purposes of this study. Every participant was identified by a code number; no names were used. If the outcome report is published, only summarized information for the entire group will be displayed.

Are you willing to take part in the research?

1. Yes 2. No

**Instruction:** Encircle or write response on space provided in front of each question

**Appendix B:** **English version questionnaires**

This questionnaire will be used to investigate the prevalence of undernutrition and its associated factors among adolescent’s girls in Diga District, Eastern Wallaga, Ethiopia, in 2023.

01. Keble: ____________________

02. House code__________________

03. Name of Data Collector: ____________ Signature__________ Date: _______

04. Name of Supervisor: ___________ ______ Signature_______ Date: _______

| **Part 1: Socio-demographic and economic characteristics** | | | | | | | | | | | | |
| --- | --- | --- | --- | --- | --- | --- | --- | --- | --- | --- | --- | --- |
| **No** | **Question** | Response | | | | | | | Skip | | | |
| 101 | Marital status of your care giver( family) | 1. Single  2. Married  3.Divorced  4.Widowed | | | | | | |  | | | |
| 102 | Household headed by: | 1. Male  2. Female | | | | | | |  | | | |
| 103 | Educational status of father? | 1. No formal education  2. primary education (1-8)  3. Secondary education (9-12)  4. College and above | | | | | | |  | | | |
| 104 | Educational status of mother? | 1. No formal education  2. primary education (1-8)  3. Secondary education (9-12)  4. College and above | | | | | | |  | | | |
| 105 | Occupation status of father’s? | 1.Farmer  2.Merchant  3.Government employer  4. Others (specify) | | | | | | |  | | | |
| 106 | Occupation status of mother’s? | 1.Farmer  2.Housewife  3.Merchant  4.Government employer  5.Others (specify) | | | | | | |  | | | |
| 107 | Family size | Specific in number__________ | | | | | | |  | | | |
| 108 | Family monthly income? | __________ETB | | | | | | |  | | | |
| 109 | Age of Adolescent girl? | _________ years | | | | | | |  | | | |
| 110 | Adolescent currently living with whom? | 1. Primary family (father & mother)  2. With Father only  3. With mother only  4.With relatives  5.With Others (specify) | | | | | | |  | | | |
| 111 | Current status adolescent girls(Job of adolescent) | 1. student  2. private work/business  3. maid  4. Others (specify) _________ | | | | | | |  | | | |
| **2.Health related factors** | | | | | | | | | | | | |
| 201 | Have you begun menstruation? | 1.Yes  2.No | | | | | | | If no, skip to question 203 | | | |
| 202 | At what age did you see your first menstruation | _________ year | | | | | | |  | | | |
| 203 | Do you have any information about nutrition and health? | 1.Yes  2.No | | | | | | | If no, skip to question 205 | | | |
| 204 | From where did you get the nutrition and health information | 1.Health professional/HEW  2. Mass media  3. Family  4.Other---------- | | | | | | |  | | | |
| 205 | Do you have previous history of disease last 2 weeks? | 1.Yes  2.No | | | | | | |  | | | |
| **Part 3: Environmental and Behavioral Factors Related questions.** | | | | | | | | | | | | |
| 301 | Source of drinking water? | 1.tap water  2.Protected spring water  3.Unprotected spring water  4.Other specify | | | | | | |  | | | |
| 302 | Do you use any water treatment methods at home? | 1.Yes  2. No | | | | | | |  | | | |
| 303 | Do you have functional latrine? | 1.Yes  2. No | | | | | | |  | | | |
| 304 | Do you wash your hands after toilet? | 1.Yes  2. No | | | | | | | If no, skip to question 401**O** | | | |
| 305 | What cleansing agents you use for washing your hand frequently? | 1.water and soap  2.water only  3.water & other traditional detergents ----------------- | | | | | | |  | | | |
| **Part 4 : Individual Dietary Diversity Score 24 Hours Diet History** | | | | | | | | | | | | |
| Now I'd want to question you about the things you ate yesterday, both throughout the day and at night (in the last 24 hours). Please put ‘’✓’’ in your answer | | | | | | | | | | | | |
| **No** | **Questions** | | | | | | | | **Yes** | | **No** | |
| 401 | Cereals such as wheat, maize, teff, sorghum, barely, and rice (injera, bread, kita, porridge, pasta, macaroni, qollo) | | | | | | | |  | |  | |
| 402 | Vitamin A rich vegetables and tubers (sweet, pumpkin, potatoes, carrots, red sweet pepper) | | | | | | | |  | |  | |
| 403 | White tubers and roots like potatoes, sweet potatoes | | | | | | | |  | |  | |
| 404 | Dark green leafy vegetables (cabbage, lettuce, spinach) | | | | | | | |  | |  | |
| 405 | Other vegetables like tomato, onion, lemon | | | | | | | |  | |  | |
| 406 | Vitamin A rich fruits like avocado, banana, papaya, peach, mango | | | | | | | |  | |  | |
| 407 | Organ meat like liver, kidney, heart | | | | | | | |  | |  | |
| 408 | Flesh meat like beef, lamb, chicken, sheep, goat | | | | | | | |  | |  | |
| 409 | Egg | | | | | | | |  | |  | |
| 410 | Fish like fresh or dried fish or shellfish | | | | | | | |  | |  | |
| 411 | Pulse (beas,peas and lentils) like telba, shiro wot,kiki | | | | | | | |  | |  | |
| 412 | Milk or milk product like yoghurt, cheese, and butter | | | | | | | |  | |  | |
| 413 | Nuts and seeds | | | | | | | |  | |  | |
| 414 | Do you use Coffee or tea between your meals? | | | | | | | |  | |  | |
| **Part 5: Food Frequency Questionnaires** | | | | | | | | | | | | |
| Now I'd want to question you about the foods you consumed in the last one month. | | | | | | | | | | | | |
| No | Questions | | Never | 1 time per month | 2-3 times per month | 1 time per week | 2 times in week | - 1. times in week | 1 time per day | 2-3 times in day | | >3 time in per day |
| 501 | How often do you consume cereal-based items such as injera, bread, porridge, pasta, and so on? | |  |  |  |  |  |  |  |  | |  |
| 502 | How often do you consume dark green vegetables such as lettuce and cabbage? | |  |  |  |  |  |  |  |  | |  |
| 503 | How frequently do you consume tubers and root vegetables such as sweet potatoes and potatoes? | |  |  |  |  |  |  |  |  | |  |
| 504 | How often do you eat vitamin A-rich vegetables and tubers (carrots, pumpkin, red sweet pepper, sweet potatoes)? | |  |  |  |  |  |  |  |  | |  |
| 505 | How often you eat fruits like banana , mango, avocado and papaya? | |  |  |  |  |  |  |  |  | |  |
| 506 | How often you eat meats? | |  |  |  |  |  |  |  |  | |  |
| 507 | How often do you eat foods produced from various sorts of eggs? | |  |  |  |  |  |  |  |  | |  |
| 508 | How often do you eat foods made from beans, lentils, or peas? | |  |  |  |  |  |  |  |  | |  |
| 509 | How often you consume milk and milk products such as yoghurt, cheese, and butter? | |  |  |  |  |  |  |  |  | |  |
| 510 | How often nuts or seeds | |  |  |  |  |  |  |  |  | |  |
| 511 | How often you use beverage like coffee and tea? | |  |  |  |  |  |  |  |  | |  |
| **Part 6 : Questions to assess household food security (HFIAS)** | | | | | | | | | | | | |
| **No** | **Questions** | **Response options (encircle one)** | | | | | | | **Skip** | | | |
| 601 | Did you worry about the household's not have enough food supply in the past four weeks? | 1=Yes  2=No | | | | | | | If no, skip Q 602 | | | |
| 601a | How often did this happen? | 1 = Rarely (1X or 2X in the past four weeks  2 = Sometimes (3x to 10x in the past four weeks)  3 = Often (>10x in the past four weeks) | | | | | | |  | | | |
| 602 | In the past four weeks, were you or any household member not able to eat the kinds of foods you preferred because of a lack of resources? | 1=Yes  2=No | | | | | | | If no, skip to Q 603 | | | |
| 602a | How often did this happen? | 1 = Rarely (1X or 2X in the past four weeks  2 = Sometimes (3x to 10x in the past four weeks)  3 = Often (>10x in the past four weeks) | | | | | | |  | | | |
| 603 | In the past four weeks, did you or any household member have to eat a limited variety of foods due to a lack of resources? | 1=Yes  2=No | | | | | | | If no, skip to Q 604 | | | |
| 603a | How often did this happen? | 1 = Rarely (1X or 2X in the past four weeks  2 = Sometimes (3x to 10x in the past four weeks)  3 = Often (>10x in the past four weeks) | | | | | | |  | | | |
| 604 | In the past four weeks, did you or any household member have to eat some foods that you really did not want to eat because of a lack of resources to obtain other types of food? | 1=Yes  2=No | | | | | | | If no, skip to Q 605 | | | |
| 604a | How often did this happen? | 1 = Rarely (1X or 2X in the past four weeks  2 = Sometimes (3x to 10x in the past four weeks)  3 = Often (>10x in the past four weeks) | | | | | | |  | | | |
| 605 | In the past four weeks, did you or any household member have to eat a smaller meal than you felt you needed because there was not enough food? | 1=Yes  2=No | | | | | | | If no, skip to Q 606 | | | |
| 605a | How often did this happen? | 1 = Rarely (1X or 2X in the past four weeks  2 = Sometimes (3x to 10x in the past four weeks)  3 = Often (>10x in the past four weeks) | | | | | | |  | | | |
| 606 | In the past four weeks, did you or any other household member have to eat fewer meals in a day because there was not enough food? | 1=Yes  2=No | | | | | | | If no, skip to Q 607 | | | |
| 606a | How often did this happen? | 1 = Rarely (1X or 2X in the past four weeks  2 = Sometimes (3x to 10x in the past four weeks)  3 = Often (>10x in the past four weeks) | | | | | | |  | | | |
| 607 | In the past four weeks, was there ever no food to eat of any kind in your household because of lack of resources to get food? | 1=Yes  2=No | | | | | | | If no, skip to Q 608 | | | |
| 607a | How often did this happen? | 1 = Rarely (1X or 2X in the past four weeks  2 = Sometimes (3x to 10x in the past four weeks)  3 = Often (>10x in the past four weeks) | | | | | | |  | | | |
| 608 | In the past four weeks, did you or any household member go to sleep at night hungry | 1=Yes  2=No | | | | | | | If no skip, to Q 609 | | | |
| 608a | How often did this happen? | 1 = Rarely (1X or 2X in the past four weeks  2 = Sometimes (3x to 10x in the past four weeks)  3 = Often (>10x in the past four weeks) | | | | | | |  | | | |
| 609 | In the past four weeks, did you or any household member go a whole day and night without eating anything because there was not enough food | 1=Yes  2=No | | | | | | | If no=questionnaire finished | | | |
| 609a | How often did this happen? | 1 = Rarely (1X or 2X in the past four weeks  2 = Sometimes (3x to 10x in the past four weeks)  3 = Often (>10x in the past four weeks) | | | | | | |  | | | |
| **Part 7 : Agricultural production related factor** | | | | | | | | | | | | |
| 701 | Do you have home gardening? | 1.Yes  2.No | | | | | | | If no, skip to Q 703 | | | |
| 702 | Purpose of home gardening? | 1.for sale  2. for consumption  3.for both | | | | | | |  | | | |
| 703 | Do you have fertile agricultural land for production? | 1.Yes  2.No | | | | | | | If 2, skip to 709 | | | |
| 704 | Ownership of the farmland | 1.Own  22.Rent | | | | | | |  | | | |
| 705 | Size of agricultural land | ______________ Hectare/s | | | | | | |  | | | |
| 706 | Annual total agricultural products (includes all items) adequate for consumption | 1.Yes  2.No | | | | | | |  | | | |
| 707 | Do you have variety of crop production? | 1.Yes  2.No | | | | | | |  | | | |
| 708 | Purpose of variety of crop production? | 1.for sale  2. for consumption  3.for both | | | | | | |  | | | |
| 709 | Do you have poultry or other animals? | 1.Yes  2.No | | | | | | |  | | | |
| **Part 8:** **Adolescent girl anthropometric measurement** | | | | | | | | | | | | |
| 801 | Weight of adolescent in | ______________Kg | | | | | | |  | | | |
| 802 | Height of adolescent in | ______________Cm | | | | | | |  | | | |

**Thank You!**

## Annex III: (Afan Oromo version).

**Participant information sheet and informed consent form for chosen participants in the study** Maqaan koo _________________ kanan jedhamu, sassaabduu odeeffannoo qo’annoo ganda keessan keessatti karaa barattuu Emabeet Booboo, barrattu digrii 2ffaa Yuniversitii Wallaga kan taateen geeggeeffamuuti. Haalumma Kanaan qoánnoo geegefamaa jiru kana keessatti hirmaattuu taatanii filatamiuu keessaniif isin galateeffachaa ibsa kennamuu akka xiyyeefanoon akka nuuf kennitaniif kabajaan isin gaafadha.

**Mata-duree qo’annichaa** -Qoánnoo Hirdhina Nyaataa fi sababoota kanaan walqabatan kan shamarran umrii 10-19tti aanaa Diigaa G/Baha Wallaga , itoophiyaa keessa jiran, bara 2023

**Kaayyoo qo’annichaa -** Kaayyoon qorannoo kanaa inni guddaan haala soorata shamarran dargaggootaa aanaa Diigaa madaaluun bu’aa qorannichaa biiroo fayyaa aanichaaf dhiyeessuun sagantaalee gidduu seensaa soorataa dargaggoota hawaasa keessatti fooyyessuu danda’an uumuuf. Kana malees, galmi qorannoo kanaa qoratuun qo’annoo kanaa digrii lammaffaa ishee fayyaa daa’immanii fi narsii fayyaa daa’immaniitiin (pediatric and child health nursing) dhaan fudhachuuf uulaagaa barbaachisu guuttachuuf ishee gargaara.

**Yeroo fudhatu fi deemsa** : Qo’annoo keenyaaf, af-gaaffii kan isiniif ni goona; akkasumas dheerinaa fi ulfaatina shammarran keessan kan saffarru yoo taú Gaaffii fi deebii godhamuuf waliigala hanga daqiiqaa 45 fudhate. Maqaan hirmaattotaa gaaffilee irratti hin mul’atu.

**miidhaa qo’anichaa fi fayyida isa** : Qorannoon kun balaa dhufuu danda'u tokkoyyuu kan hin qabnee fi yeroo keessan keessaa daqiiqaa 45 qofa fudhata. Argannoon qorannichaa karoorfattoonni fayyaa( health planner ) naannoo fi aanaa akka madda odeeffannootti kan itti fayyadaman ta'a.

**Iccitii eeguu:** Odeeffannoon hundi iccitii ta'ee kan eegame yoo ta'u, qaama sadaffaa kamiifuu dabarsamee hin kennamu. Maqaan hirmaattotaa akka hin beekamneef waraqaa gaaffii irratti hin galmaú; gareen qorannoo qofti odeefanicha argachuu danda’a.

**Mirga**: Hirmaattonni odeeffannoo laachuu dhiisuf mirga guutuu qabu. Hirmaannaan guutummaatti fedhii ofiitiin kan raawwatamudha.

**Teessoo:-**Haala qorannicha ilaalchisee gaaffii, qeeqa ykn yaada yoo qabaattan kara email emebetbobob2008@gmail.com ykn Lakk. bilbilaa +251911126654 qoa’tuu Imabeet Booboo qunnauu ni dandeessu. Kanaffuu qorannoo kanaa kessattii hirmachuuf feedhii qabduu?

1.Eyyee 2. lakkii

**Gallattoma!!**

**Appendix B:** **Afan Oromo version questionnaires**.

Gaaffileen qorrannoo armaan gaditti dhiyaatan babaldhina hirdhina nyaataaf rakkowwan walqabatanni shamarranii umurii 10-19tti jiran, baha wallaggaa , aanaa Diggaa keessatti aragman iirratti muldhatu qorachhuf qopha’e. Bara 2023.

1. Ganda: _______________________

02. Lakk. manaa_________________

03. Maqaa raga sassaabduu: ________________ Mallattoo___________ Guyyaa: ________

04. Maqaa to’ataa/ttuu: _____________________ Mallattoo___________ Guyyaa:________

| **Kuta 1ffaa :** Gaafiwwan dhimma Hawaasa fi dinagdeen wal qabatan | | | | |
| --- | --- | --- | --- | --- |
| **Lak** | **Gaaffii** | | **Deebii** | **Yaada** |
| 101 | Halla fudha fi heruma maatii | | 1. walfudhanii kan waliin jirracha jiran |  |
|  |  |  | 2. Abbaan warraa kan irraa du’e |  |
|  |  |  | 3. Kan hike/tte |  |
|  |  |  | 4. Kan biro |  |
|  |  |  | 5. Kan birroo--------- |  |
| 102 | Dura bu’a maatii eenyu? | | 1.Dhirra  2. Dhala |  |
| 103 | Sadarkaa barumsaa abbaa kee maalii? | | 1.mana barumsa kan hin galle |  |
|  |  |  | 2.Sadrkaa tokkofa (1-8) |  |
|  |  |  | 3. Sdarkaa 2ffaa (9-12) |  |
|  |  |  | 4. Koollojjii/universiitii |  |
| 104 | Sadarkaan barumsaa haadha kee mallei? | | 1.mana barumsa kan hin galle |  |
|  |  |  | 2.Sadrkaa tokkofa (1-8) |  |
|  |  |  | 3. Sdarkaa 2ffaa (9-12) |  |
|  |  |  | 4. Koollojjii/universiitii |  |
| 105 | Dalagaan abbaake maalii? | | 1. Qoteebulaa |  |
|  |  |  | 2. Daldalaaa |  |
|  |  |  | 3. Hojjataa motummaa |  |
|  |  |  | 4. Kan biroo(ibsi)____ |  |
| 106 | Dalagaan haadhakee maalii? | | 1.Qoteebulaa |  |
|  |  |  | 2.Hadhaa warraa |  |
|  |  |  | 3.Daldalaa |  |
|  |  |  | 4.Hojetaa motummaa |  |
|  |  |  | 5. Kan biro---------- |  |
| 107 | Bayyini maati kessaanii waliin jiraattaan lakk. Meeqa? | | ------------- |  |
| 108 | Galiin maattii kessannii tilmamaan ji’atti qarshii meeqa(ETB) | | ---------------- Birr |  |
| 109 | Umuri kee waggaa meeqa(shammarree) | ------------ waggaa | |  |
|  |  |  | |  |
| 110 | Yeroo amma kana eenyu waliin jiraachaa | 1. Abba fi Haadhakoo waliin | |  |
|  |  | 2. Abba koo waliin | |  |
|  |  | 3. Hadha koo waliin | |  |
|  |  | 4. Fira koo waliin | |  |
|  |  | 5. kan biroo | |  |

| 111 | Haala yeroo amman kana shamarreen irra jirtu(hojii) | 1. Barattuu |  |
| --- | --- | --- | --- |
|  |  | 2.. Hojii dhuunfaa/Biznesii |  |
|  |  | 3. Hojjettu mana nama |  |
|  |  | 4.Kan biro |  |
| **2^ffaa.^Gaaffiwwan Fayyaa waliin walqabatan** | | | |
| 201 | Laguu ji’a arguu eegalteettaa ? | 1. Eeyyen |  |
|  |  | 2. Lakkii | 2 yoo ta’e gar gaaffii 603 ti darbi |
| 202 | Umurii kee meeqatti yeroo jalqabaaf lagu ji’a argite? | Waggaa ................tti |  |
| 203 | Odeeffannoo fayyaa sirna nyaata walqabtee dhageessee/hirmaatee beekattaa? | 1. Eeyyen |  |
|  |  | 2. Lakkii |  |
| 204 | Odeeffannicha eessaa argatte?  . | 1.Hojjetootta fayyaa irraa /HEW |  |
|  |  | 2.Miidiyaa Sabqunamtii irraa |  |
|  |  | 3.Maatii irraa |  |
|  |  | 4.Kan biroo |  |
| 205 | Torban lamman darbban keessa dhukkubbaa kamiyu dhukkubsatee turttee? | 1. Eeyyen |  |
|  |  | 2. Lakki |  |

| **Kuta 3ffaaa : Wa’ee nannoo fi amaloota waliqabataan** | | | | | | | | | | | | | | | | | |
| --- | --- | --- | --- | --- | --- | --- | --- | --- | --- | --- | --- | --- | --- | --- | --- | --- | --- |
| 301 | | Maadda bishaan dhugatii kessaan? | | 1.Bishaan bombaa | | | | | | | |  | | | | | |
|  |  |  |  | 2.Burqitu ijaarsaan eegamaa | | | | | | | |  | | | | | |
|  | |  | | 3.Burqituu ya’aa ijaarsa ala/ hinqabne | | | | | | | |  | | | | | |
|  |  |  |  | 4.Kan biro(ibsi)………. | | | | | | | |  | | | | | |
| 302 | | Tofta qulqullina bishaan itti eegan ni fayyadamttuu? | | 1.Eeyeen | | | | | | | |  | | | | | |
|  |  |  |  | 2.Lakki | | | | | | | |  | | | | | |
| 303 | | Mana fincaanii itti fayadamaa jirtan qabduu/jiraa? | | 1.Eeyyen | | | | | | | |  | | | | | |
|  |  |  |  | 2. Lakki | | | | | | | |  | | | | | |
| 304 | | Mana fincaanii erga fayyadamte booda harka keen ni dhiqattaa? | | 1..Eeyyen | | | | | | | |  | | | | | |
|  |  |  |  | 2. Lakki | | | | | | | |  | | | | | |
| 305 | | Halli harka dhiqana kee /Harka kee dhiqachuuf maal fayyadamtta? | | 1.Samunaa fi Bishaan | | | | | | | |  | | | | | |
|  |  |  |  | 2.Bishaan qofaa | | | | | | | |  | | | | | |
|  |  |  |  | 3.Kan biro(Ibsii-----) | | | | | | | |  | | | | | |
|  | |  | | | | | | | | | | | | | | | |
|  | | **Kutaa 4^ffaa^. Gaafiwwan soorata /nyaata sa’aatii 24 keessat (guyyaa 1 ) waliin wal qabatu** | | | | | | | | | | | | | | | |
| Gaaffiwwan armaan gadditti dhiyaatan gosoota soorata ati kaleessaa guyyaa fi galgala nyaatte (sa’a 24 darbe keessatti). Gosoota sooraata tarreefaman keessa tokkollee yoo sooratte/ nyaatte ‘’eeyye’’ tokkollee kan hin nyaanne yoo taémoo ‘’lakki ‘’jechuun deebiikee maallatto ‘’ ✓ ‘’ Kanaan bakka buusi. | | | | | | | | | | | | | | | | | |
| **Lakk** | | **Akakuu nyaataa soorattee** | | | | | | | | | | **Eeyyen** | | | **Lakki** | | |
| 401 | | Buddeena Daabboo, pasta, , mokoroni, marqaa kita, akaayii, , ,ykn gosoota soorataa midhaan dheedhii naannotti omishaman kan akka; taafii , qamadii,ruuzi , boqollo, garbuu, mishingaa, , fi k.k.f. Irraa tolfaman | | | | | | | | | |  | | |  | | |
| 402 | | Nyatoota vitamin A badhadhan kaneen akka hundee dimaa ,buuqee/dabaaqula ,karrotti… | | | | | | | | | |  | | |  | | |
| 403 | | Gosoota soorataa hiddi isaani nyaatamu kanneen akka Mosee/Dinnicha, boínaa/mixaaxisii,goodaree, kkf… | | | | | | | | | |  | | |  | | |
| 404 | | Soorataa Kuduraalee baala magarisaa taán akka raafuu/goommana, salaaxaa, raafuu maramaa, qoosxaa,…kkf | | | | | | | | | |  | | |  | | |
| 405 | | Gosoota Kuduraalee fi fuduraalee kan biro kanneen akka qullubbii , timaatimii, burtukaana, loomii, kkf | | | | | | | | | |  | | |  | | |
| 406 | | Soorata Fuduraalee vitaamina ‘A’n badhaadhan kan akka maangoo, avokaadoo, muuzii, pappaayyaa, ykn juusa/cuunfaa fuduraalee kana irraa tolfaman. | | | | | | | | | |  | | |  | | |
| 407 | | Gsoota nyaataa Foon horii fi qaamole kan akka onnee ,tiruu, kkf irra tolfaman | | | | | | | | | |  | | |  | | |
| 408 | | Flesh meat like beef, lamb, chicken, sheep, goat, | | | | | | | | | |  | | |  | | |
| 409 | | Hanqaaquu/killee | | | | | | | | | |  | | |  | | |
| 410 | | Qurxummii/sardiinii | | | | | | | | | |  | | |  | | |
| 411 | | Soorat /itto kan missira,baaqela,atara ,kiki. eto shiro fi akkasumas kan akka telba, lawziif kkf irra tolfaman | | | | | | | | | |  | | |  | | |
| 412 | | Aannanii fi buálee/oomisha aannanii kan akka Itittuu, baaduu/ayibi fi dhadhaa kkf | | | | | | | | | |  | | |  | | |
| 413 | | Nyaata zayitaa kan akkaa loozii ykn onchooloni, nuugii,talbaa, suufii.kkf | | | | | | | | | |  | | |  | | |
| 414 | | Osoo nyaata nyaacha jirtuu waan ho’aa kan akka buna shayii giddutti ni dhugda? | | | | | | | | | |  | | |  | | |
|  | |  | | | | | | | | | |  | | |  | | |
| **Kuta 5ffaa :Irra deddebii gartulee nyaata kan ji’a tokko derbee** | | | | | | | | | | | | | | | | | |
| **Gosoota soorataa/nyaataa tarrefamnii jiran keessaa jiá tokkon (guyyoota 30n) darban keessatti yeroo maaeqaaf akka nyaatan deebisaa. Deebiin ati gaaffiwwan armaan gaditti tareefamanii jiran filattu guyyaa tokko keessatti garee soorataa tokko keesaa gosa nyaata tokkoof isaa ol soortatte akka waan gosa soorataa sana nyaateetti fudhatam.**  **Bakka deebii keetti mallattoo ‘’✓’’** | | | | | | | | | | | | | | | | | |
| **Lakk** | | **Akkaakuu Nyaataa** | | | **Hin nyanne** | | **Ji’atti tokko**  **atti tokko** | **Ji’atti 2-3** | **Torbanitti 1** | | **Torbanitti 2** | | **Torbanitti 3-4** | **Guyya guyyan** | **Guyyatti 2-3** | | **Guyyatti si’aa3 ol** |
| 501 | | Gosoota Midhaan dheedhii/calla irraa tolfaman kan akka buddeena, daabboo, marqa fi pasta fi ynatawaan biro daaguzzaa, mishingaa/ boobee,xaaffi ,boqolloo ,ruzii,qamadii irraa qophaa’an? | | |  | |  |  |  | |  | |  |  |  |  | |
| 502 | | Kuduraalee ballisaan magaarisa garaa gurachaa ta’aan kan akka raaffuu,qaara fi kuduraa ballisaa magarisaa kan biroo? | | |  | |  |  |  | |  | |  |  |  |  | |
| 503 | | Gosoota sooraataa kan hiddi isaanii nyaatamu kanneen akka mixaaxisii/boína, dinicha, mosee ancootee fi kkf irraa qopha’ani? | | |  | |  |  |  | |  | |  |  |  |  | |
| 504 | | Fuduraalee kan akka pappaayyaa, maangoo, avokaadoo fi muuzii irraa qophaa’e nyatu? | | |  | |  |  |  | |  | |  |  |  |  | |
| 505 | | Foon( foonii loonii, hoollaa, reetti fi lukkuu) kanneen akka lukku , tiruu ,kale fi , onnee nyatu? | | |  | |  |  |  | |  | |  |  |  |  | |
| 506 | | hanqaaquu/killee nyatu? | | |  | |  |  |  | |  | |  |  |  |  | |
| 507 | | Qurxummii/sardiinii nyatu? | | |  | |  |  |  | |  | |  |  |  |  | |
| 508 | | Gosoota Nyaata kanneen baqela, missira, fi atara irraa hojjeteme nyatu? | | |  | |  |  |  | |  | |  |  |  |  | |
| 509 | | Bu’alee Aannan fi omisha aannanii kan taꞌan kan akka itittu, baaduu/ ayibe, fi dhadhaa nyatu? | | |  | |  |  |  | |  | |  |  |  |  | |
| 510 | | Nyaata zayitaa kan ta’aan kan akka lowzii,oncholoni,nuugi,talbaa | | |  | |  |  |  | |  | |  |  |  |  | |
| 511 | | Buna/shayii | | |  | |  |  |  | |  | |  |  |  |  | |
|  | | | | | | | | | | | | | | | | | |
| **Kuta 6^ffaa^: Gaaffiilee haala wabii nyaataa ilaallatan** | | | | | | | | | | | | | | | | | |
| **Lakk.** | | **Gaaffiwwan** | **Fillanowwan kee**  **ittii mari.** | | | | | | | | | **Irra darbi** | | | | | |
| 601 | | Torbanoota 4n darbanii jiran keessatti maatiin keessan nyaata gahaa hin qabu ykn soorati jalaa dhuma jechuun yaaddooftanii turtanii? | 1. Eeyyeen | | | | | | | | | 1 yoo ta’e gar gaaffii 602 ti darbi | | | | | |
|  |  |  | 2. Lakki | | | | | | | | |  |  |  |  |  |  |
| 601a | | Yeroo jedhame keessatti siá meeqaaf yaadoon kun isin mudatee jira? | 1. Yeroo xiqoof (jiá darbetti al 1- 2 tti) | | | | | | | | |  | | | | | |
|  |  |  | 2.Al tokko tokko (jiá darbetti al 3- 10 tti) | | | | | | | | |  | | | | | |
|  |  |  | 3. Yeroo baýyeef ( jiá darbetti yeroo 10 nii ol) | | | | | | | | |  | | | | | |
| 602 | | Torbaanoota 4n darban keessatti miseensi matii keessaniis taé isiin maalaqa dhabuun soorata filattan osoo hin nyaatiin haftanii jirtuu? | 1. Eeyyeen | | | | | | | | | 2 yoo ta’e gar gaaffii 603 ti darbi | | | | | |
|  |  |  | 2. lakki | | | | | | | | |  |  |  |  |  |  |
| 602a | | Hanqin ykn Rakkoon armaan olii kun yoo jiraate siá meeqa isin quuname? | 1. Yeroo xiqoof (jiá darbetti al 1- 2 tti) | | | | | | | | |  | | | | | |
|  |  |  | 2.Al tokko tokko (jiá darbetti al 3- 10 tti) | | | | | | | | |  | | | | | |
|  |  |  | 3. Yeroo baýyeef ( jiá darbetti yeroo 10 nii ol) | | | | | | | | |  | | | | | |
| 603 | | Torbaanoota 4n darban keessatti miseensi matii keessaniis taé isiin maalaqa dhabuun gosoota soorataa muraasa qofaa nyaachuuf yeroon isin itti dirqamtan jira turee? | 1. Eeyyeen | | | | | | | | | 2 yoo ta’e gar gaaffii 604 ti darbi | | | | | |
|  |  |  | 2. lakki | | | | | | | | |  | | | | | |
| 603a | | Yeroo jedhame keessatti siá meeqaaf rakkoon kun isin mudatee jira? | 1. Yeroo xiqoof (jiá darbetti al 1- 2 tti) | | | | | | | | |  | | | | | |
|  |  |  | 2.Al tokko tokko (jiá darbetti al 3- 10 tti) | | | | | | | | |  | | | | | |
|  |  |  | 3. Yeroo baýyeef ( jiá darbetti yeroo 10 nii ol) | | | | | | | | |  | | | | | |
| 604 | | Torbaanoota 4n darban keessatti miseensi matii keessaniis taé isiin maalaqa dhabuun nyaata nyaachuu hin feene/hinbarbaan nyaachuuf yeroon itti dirqamtan jira turee? | 1. Eeyyen | | | | | | | | | 2 yoo ta’e gar gaaffii 605 ti darbi | | | | | |
|  |  |  | 2. Lakkii | | | | | | | | |  |  |  |  |  |  |
| 604a | | Yeroo jedhame kana keessatti rakkoon kun siá meeqaaf isin mudatee jira? | 1. Yeroo xiqoof (jiá darbetti al 1- 2 tti) | | | | | | | | |  | | | | | |
|  |  |  | 2.Al tokko tokko (jiá darbetti al 3- 10 tti) | | | | | | | | |  | | | | | |
|  |  |  | 3. Yeroo baýyeef ( jiá darbetti yeroo 10 nii ol) | | | | | | | | |  | | | | | |
| 605 | | Torbaanoota 4n darban keessatti miseensi matii keessaniis taé isiin nyaanni gahaan wan dhibeef nyaata hanga sorachuu barbaaddaniin gaditti nayaachuuf yeroon itti dirqamtan jira turee? | 1. Eeyyen | | | | | | | | |  | | | | | |
|  |  |  | 2. Lakkii | | | | | | | | | 2 yoo ta’e gar gaaffii 606 ti darbi | | | | | |
| 605a | | Yeroo jedhame kana keessatti rakkoon kun siá meeqaaf isin mudatee jira? | 1. Yeroo xiqoof (jiá darbetti al 1- 2 tti) | | | | | | | | |  | | | | | |
|  |  |  | 2.Al tokko tokko (jiá darbetti al 3- 10 tti) | | | | | | | | |  | | | | | |
|  |  |  | 3. Yeroo baýyeef ( jiá darbetti yeroo 10 nii ol) | | | | | | | | |  | | | | | |
| 606 | | Torbaanoota 4n darban keessatti miseensi matii keessaniis taé isiin nyaanni gahaan wan dhibeef guyyaatti yeroo muraasa qofa soorachuuf yeroon itti dirqamtan jira turee? | 1. Eeyyen | | | | | | | | |  | | | | | |
|  |  |  | 2. Lakkii | | | | | | | | | 2 yoo ta’e gar gaaffii 607 ti darbi | | | | | |
| 606a | | Yeroo jedhame kana keessatti rakkoon kun siá meeqaaf isin mudatee jira? | 1. Yeroo xiqoof (jiá darbetti al 1- 2 tti) | | | | | | | | |  | | | | | |
|  |  |  | 2.Al tokko tokko (jiá darbetti al 3- 10 tti) | | | | | | | | |  | | | | | |
|  |  |  | 3. Yeroo baýyeef ( jiá darbetti yeroo 10 nii ol) | | | | | | | | |  | | | | | |
| 607 | | Torbaanoota 4n darban keessatti miseensi matii keessaniis taé isiin maalaqa dhabuun gosa soorataa kamiyyuu mana keessaa yeroon itti dhabdan tureeraa? | 1. Eeyyen | | | | | | | | | 2 yoo ta’e gar gaaffii 608 ti darbi | | | | | |
|  |  |  | 2. Lakkii | | | | | | | | |  |  |  |  |  |  |
| 607a | | Yeroo jedhame kana keessatti rakkoon kun siá meeqaaf isin mudatee jira? | 1. Yeroo xiqoof (jiá darbetti al 1- 2 tti) | | | | | | | | |  | | | | | |
|  |  |  | 2.Al tokko tokko (jiá darbetti al 3- 10 tti) | | | | | | | | |  | | | | | |
|  |  |  | 3. Yeroo baýyeef ( jiá darbetti yeroo 10 nii ol) | | | | | | | | |  | | | | | |
| 608 | | Torbaanoota 4n darban keessatti miseensi matii keessaniis taé isiin nyaanni gahaan dhabuun beela’aa ykn garaa duwwaa ciisuuf yeroon itti dirqamtan jira turee? | 1. Eeyyen | | | | | | | | | 2 yoo ta’e gar gaaffii 609 ti darbi | | | | | |
|  |  |  | 2. Lakkii | | | | | | | | |  |  |  |  |  |  |
| 608a | | Yeroo jedhame kana keessatti rakkoon kun siá meeqaaf isin mudatee jira? | 1. Yeroo xiqoof (jiá darbetti al 1- 2 tti) | | | | | | | | |  | | | | | |
|  |  |  | 2.Al tokko tokko (jiá darbetti al 3- 10 tti) | | | | | | | | |  | | | | | |
|  |  |  | 3. Yeroo baýyeef ( jiá darbetti yeroo 10 nii ol) | | | | | | | | |  | | | | | |
| 609 | | Torbaanoota 4n darban keessatti miseensi matii keessaniis taé isiin nyaanni gahaa dhabuutiin galgalaa fi guyyaa guutuu beela’aa ykn garaa duwwaa yeroon itti turuuf dirqamtan jira turee? | 1. Eeyyen | | | | | | | | |  | | | | | |
|  |  |  | 2. Lakkii | | | | | | | | | 2= gaaffiin dhumeeraa | | | | | |
| 609a | | Yeroo jedhame kana keessatti rakkoon kun siá meeqaaf isin mudatee jira? | 1. Yeroo xiqoof (jiá darbetti al 1- 2 tti) | | | | | | | | |  | | | | | |
|  |  |  | 2.Al tokko tokko (jiá darbetti al 3- 10 tti) | | | | | | | | |  | | | | | |
|  |  |  | 3. Yeroo baýyeef ( jiá darbetti yeroo 10 nii ol) | | | | | | | | |  | | | | | |
|  | | | | | | | | | | | | | | | | | |
|  | |  | | | |  | | | | | |  | | | | | |
| **Kutaa 7^ffaa^: Gaffille Oomisha midhaan nyaattaa wallin walqabataan** | | | | | | | | | | | | | | | | | |
| **Lakk.** | **Gaaffiwwan** | | | | | **Deebii** | | | | | | | | | | | |
| 701 | | Attakilitin mora manaa kessani jira ? | | | | 1.Eeyye | | | |  | | | | | | | |
|  |  |  |  |  |  | 2.Lakki | | | |  | | | | | | | |
| 702 | | Attakilitti omishtaan mallif olchituu? | | | | 1. Gurgurtaaf | | | |  | | | | | | | |
|  |  |  |  |  |  | 2. Nyataaf | | | |  | | | | | | | |
|  |  |  |  |  |  | 3.Nyataafi gurgurtaaf | | | |  | | | | | | | |
| 703 | | Lafa qonnaaamma irraa omishaa argachaa jirtan qabduu? | | | | 1.Eeyye | | | | 2 yoo ta’e gar gaaffii 709 ti darbi | | | | | | | |
|  |  |  |  |  |  | 2.Lakki | | | |  | | | | | | | |
| 704 | | Abban qabiyyee lafa qonnichichaa eenyu? | | | | 1.keenya | | | |  | | | | | | | |
|  |  |  |  |  |  | 2. Kirayii | | | |  | | | | | | | |
| 705 | | Baldhina lafa qonnaa | | | | Hetaaran---------- | | | |  | | | | | | | |
| 706 | | Omishaa qonaa waligalaa kan waagaan argataan nyataa matti kessannif gahadhaa | | | | 1.Eyyee  2.lakki | | | |  | | | | | | | |
| 707 | | Gosa midhaan nyaataa garagarra ni omishituu | | | | 1.Eeyyee | | | |  | | | | | | | |
|  |  |  |  |  |  | 2 .lakki | | | |  | | | | | | | |
| 708 | | Gosa midhaan nyaataa garagarra ni omishitaan mallif olchitu? | | | | 1.Gurgurtaf | | | |  | | | | | | | |
|  |  |  |  |  |  | 2. Nyataaf | | | |  | | | | | | | |
|  |  |  |  |  |  | 3.Nyataafi gurgurtaaf | | | |  | | | | | | | |
| 709 | | Qabiyee dhunfaa kan maatii ta’e Bushayee, Sawwan fi /ykn Lukku qabduu? | | | | 1.Eeyyee | | | |  | | | | | | | |
|  |  |  |  |  |  | 2 .lakki | | | |  | | | | | | | |
|  | |  | | | |  | | | | | |  | | | | | |
| **Kutaa 8^ffaa^: Safarrii haanga guddina shamarree ibsu** | | | | | | | | | | | | | | | | | |
| 801 | | Ulfaatina shammarre | | | | Kilograma(Kg)_______ | | | | | | | | | | | |
| 802 | | Dheerinaa shammarre | | | | Sentimeetira (Cm)_____________ | | | | | | | | | | | |

| **Galatoomaa!!** |
| --- |
